# K-Means Spreading Factor Allocation for Large-Scale LoRa Networks

**DOI:** 10.3390/s19214723

**Published:** 2019-10-30

**Authors:** Muhammad Asad Ullah, Junnaid Iqbal, Arliones Hoeller, Richard Demo Souza, Hirley Alves

**Affiliations:** 1 Centre for Wireless Communications, University of Oulu, 90014 Oulu, Finland; muhammad.asadullah@oulu.fi (M.A.U.); junnaid.iqbal@oulu.fi (J.I.); 2 Department of Electrical and Electronics Engineering, Federal University of Santa Catarina, Florianópolis 88040-900, Brazil; richard.demo@ufsc.br; 3 Department of Telecommunications Engineering, Federal Institute for Education, Science, and Technology of Santa Catarina, São José 88103-310, Brazil

**Keywords:** stochastic geometry, resource allocation, Internet of Things

## Abstract

Low-power wide-area networks (LPWANs) are emerging rapidly as a fundamental Internet of Things (IoT) technology because of their low-power consumption, long-range connectivity, and ability to support massive numbers of users. With its high growth rate, Long-Range (LoRa) is becoming the most adopted LPWAN technology. This research work contributes to the problem of LoRa spreading factor (SF) allocation by proposing an algorithm on the basis of K-means clustering. We assess the network performance considering the outage probabilities of a large-scale unconfirmed-mode class-A LoRa Wide Area Network (LoRaWAN) model, without retransmissions. The proposed algorithm allows for different user distribution over SFs, thus rendering SF allocation flexible. Such distribution translates into network parameters that are application dependent. Simulation results consider different network scenarios and realistic parameters to illustrate how the distance from the gateway and the number of nodes in each SF affects transmission reliability. Theoretical and simulation results show that our SF allocation approach improves the network’s average coverage probability up to 5 percentage points when compared to the baseline model. Moreover, our results show a fairer network operation where the performance difference between the best- and worst-case nodes is significantly reduced. This happens because our method seeks to equalize the usage of each SF. We show that the worst-case performance in one deployment scenario can be enhanced by 1.53 times.

## 1. Introduction

The Internet of Things (IoT) is the integration of modern electronic devices, smart sensors, internet protocols, and wireless communications technologies. IoT applications are rapidly gaining popularity in many domains such as industrial operations, smart parking, augmented maps, healthcare, smart cars, and smart homes [1,2,3,4,5]. According to a Gartner Inc. report, there will be around 26 billion IoT devices deployed worldwide by 2020 [6]. In the Statista report, it is predicted that there will be over 75 billion IoT devices worldwide by 2025 [7].

In the modern era, the spectacular growth and transformation of wireless connectivity are driven by the IoT paradigm, with technologies having attributes of large-scale network infrastructure with low-cost sensors connected to the Internet. In this context, low-power wide-area networks (LPWANs) are quite popular in terms of prototypes, standards, and on the commercial level because of their significance with respect to power efficiency along with long range [8,9]. Within this context, LoRA, SigFox, NB-IoT, Weightless, RPMA and DASH7 [10,11] are the most distinguished technologies.

This paper focuses on LoRa, which provides good performance in terms of reliability and energy consumption. The network architecture contains end-devices, gateways, and a network server (NS), forming a star topology. It operates at unlicensed frequency ISM (Industrial, Scientific, Medical) bands of 863–870 MHz and 915 MHz in Europe and the U.S., respectively [12,13]. In Europe, the duty cycle limitations range from 0.1% to 10%, following European Telecommunications Standards Institute (ETSI) standards. In addition, LoRa works on variable and adaptive data rates by using different spreading factors. This is achieved by the NS controlling the spreading factors (SFs) and bandwidth (BW) of the end-devices. Higher SFs allow larger coverage areas; however, as a drawback, they reduce the data rate and increase the time-on-air (ToA) of LoRa packets [14].

Notably, the gateway has the ability to receive data from multiple nodes at the same time because of the orthogonality of sub-bands and the quasi-orthogonality of different SFs. The LoRa MAC layer, known as LoRaWAN [15], is a type of ALOHA protocol controlled by the NS. LoRaWAN defines three classes of devices depending upon the application. Class A devices may wait for acknowledgments (ACK) only in their receiving windows during downlink transmission and consume the least power. Class B devices are able to open extra receiving windows at scheduled times, thus reducing downlink latency. Class C nodes consume the most energy because they leave the receiver enabled all the time, allowing for the lowest latency time [16].

For instance, extensive measurement campaigns show that the communication range of LoRa reaches up to 30 km over the water and more than 15 km on the ground [11]. LoRa is suitable for a wide range of telemetry applications (e.g., sensing and monitoring), which can be used in several industry verticals, such as smart grids and cities, and smart agriculture up to industrial IoT applications [17,18]. During the past few years, many studies have contributed by proposing new algorithms, systems models, analyses, and by designing new approaches for performance enhancement of LoRa networks. However, only a few considered resource allocation.

The major contribution of this work is the modeling of an approach for SF allocation for a large scale LoRa network based on K-means clustering and the analysis of connection, capture, and coverage probabilities. Instead of using constant steps of distance from the gateway to define SF areas [19,20], the proposed algorithm assigns a maximum range of individual SF regions, which allows for distinct user distribution. Then, we evaluate the performance of the proposed algorithm over the uplink of a large-scale LoRa network with a single gateway based on the model introduced in [19].

The remainder of this article is structured as follows. Section 2 discusses related work and a short overview of LPWAN technologies. Section 3 introduces the system model, and Section 3.1 details the outage probabilities of the baseline model, used to examine the performance of proposed SF allocation approach. The proposed algorithm is presented in Section 4. Simulation results are discussed in Section 5. Finally, Section 6 concludes the paper and proposes future work.

## 2. Related Work

Overviews of LoRa and LPWAN technologies are provided in [21,22]. Usually, LoRa operates with a bandwidth of 125 kHz, but it also allows for bandwidths of 250 kHz and 500 kHz. The wider bands promote resistance to fading, channel noise, Doppler effects, and long-term relative frequency [23]. Chirp spread spectrum (CSS) modulation, which enables high receiver sensitivity, makes LoRa more robust against the interference when compared to Sigfox, which employs ultra-narrowband (UNB) communication [24]. As a tradeoff, the use of wider bands for the transmission of narrowband signals makes less efficient use of the spectrum. A realistic SigFox communication model is implemented and tested in [25]; it evaluates the performance of a high-density large-scale wireless sensor network (WSN). From the obtained results, one can observe that the performance of the SigFox network significantly degrades by increasing the number of sensors, and some solutions are presented to improve the performance.

Unlike Sigfox, LoRa can be deployed locally, i.e., without the need for a cellular infrastructure, and has higher bit rates. By contrast, NB-IoT is an expensive technology having the pros of low latency and high quality of service (QoS) [26]. In [27], the authors compare different LPWAN technologies (Bluetooth, ZigBee, SigFox, and LoRa) and discuss LoRa with respect to code rate (CR), bandwidth (BW), and SF but without considering the influence of Rayleigh fading and path loss attenuation. Theoretical and simulation results show that SF, BW, and CR influence the ToA of a packet. Larger SFs and CRs result in higher ToA of LoRa packets. Conversely, ToA reduces with larger bandwidths.

The work in [28] proposes two different algorithms named EXPLoRa-SF and EXPLoRa-AT and shows in simulation results that these algorithms perform considerably better than the LoRaWAN adaptive rate strategy (ADR). EXPLoRa-AT delivers higher bit rates in the event of higher traffic loads, while EXPLoRa-SF allocates SFs at the different subgroups of end-devices depending on the received signal strength indicator (RSSI). The results demonstrate that the data extraction rate (DER) drops dramatically for higher SFs and larger numbers of end-devices. The authors, however, assume a short range and dense network in their analysis.

The EXPLoRa approach is further extended in [29], K-means is applied to identify the non-circular crowded region, and all the nodes inside that area are assumed to have same SF. On the other hand, in the proposed work the geometry of network is circular, with six annuluses representing the range of individual SFs. We have analyzed the scalability and the performance of the uplink LoRa model considering Rayleigh fading, connection $H_{1}$, capture $Q_{1}$, and coverage probabilities $H_{1}$$Q_{1}$ in the presence of interfering signals using the same SF. The considerations of $H_{1}$ and $H_{1}$$Q_{1}$ are missing in [28,29]. Moreover, in our model, we consider a dense and wide network (radius of several kilometers) and analyze the performance by considering the maximum distance of individual SF boundaries from the gateway.

Another scientific study used K-means for the classification of end-devices into three groups based on traffic characteristics with different priorities. The grouping of end-devices was computed in terms of priority-based transmission instead of SF allocation [30].

In [31,32], SF distribution is mainly based on the power level of the signals that the gateway receives from the end-devices and gateway sensitivity, without considering the location of end-devices. As a drawback, SF allocation was disturbed because of high-density buildings, and 53.2% of the end-devices were forced to use SF12. Furthermore, in [28,29,30,31], only network-level simulators such as ns-3 and LoRaSim are used, which abstracts some characteristics of the physical layer that are incorporated in our analysis. Conversely, our study evaluated the performance of the proposed SF allocation algorithm considering the analytical model, realistic parameters, and averaging over $10^{5}$ random deployment of the Poisson point process (PPP) by Monte Carlo computer simulations, which match with the theoretical results.

The tree-based spreading factor clustering algorithm (TSCA) for SF allocation in multihop LoRA networks is introduced in [33]. This approach offloads the data traffic in many sub-networks, which are linked to a sink node assigning a specific SF according to network clustering, thus enabling parallel frame transmission with multiple SFs. The authors show that TSCA increases the network performance in a network with rectangular geometry.

A single gateway uplink model considering path loss attenuation and Rayleigh fading is designed in [19], utilizing stochastic geometry to model network interference and then disconnection and collision probabilities. Such a model is further extended in [20], in which the authors propose a scheme that considers message replication and gateways with multiple receive antennas/decoders to attain time and spatial diversity. They demonstrate that the number of users and traffic density directly affects the performance of the LoRa network and that sending multiple message copies is beneficial for low-density networks. Both of these studies adopt equal radius SF allocation approaches. Unlike [19,20], our work considers K-means-based fair SF allocation of nodes in LoRa networks.

Recently, several studies have addressed the problems associated with automatic repeat request (ARQ) and contributed to downlink reliability in LoRaWAN applications. The sequential transmission of downlink frames, saturation of duty cycle, and half-duplex nature of LoRa gateway radios are marked as the major shortcomings for the downlink transmission [34,35]. Furthermore, these works also highlight the significance of gateway selection algorithm to prevent traffic losses due to sequential transmission of downlink frames and duty cycle limitations.

One experimental study evaluates the performance of a LoRa network at a 125 kHz bandwidth and SF7 for a sailing monitoring model, and the measurements show a 60.49% packet loss at the maximum distance of 3284 m [36]. Another LoRaWAN-based indoor environment monitoring system composed of 331 sensor nodes is deployed at the University of Oulu, where the gateway is installed at a distance of ∼180 m and 24 m above the ground [37]. The measurements performed at SF7 show a maximum 11.33% packet error rate (PER), which can be due to co-spreading factor interference because all 331 end-devices use the same SF. As illustrated in [19], nodes using the same SF face co-spreading factor interference. The motivation behind our work is to propose a suitable SF allocation algorithm for a large-scale LoRa network to efficiently utilize the different data rates. To enhance SF allocation, we propose a novel algorithm, based on the machine learning technique called K-means clustering, for effectively allocating the SFs.

## 3. System Model

**Figure 1:**
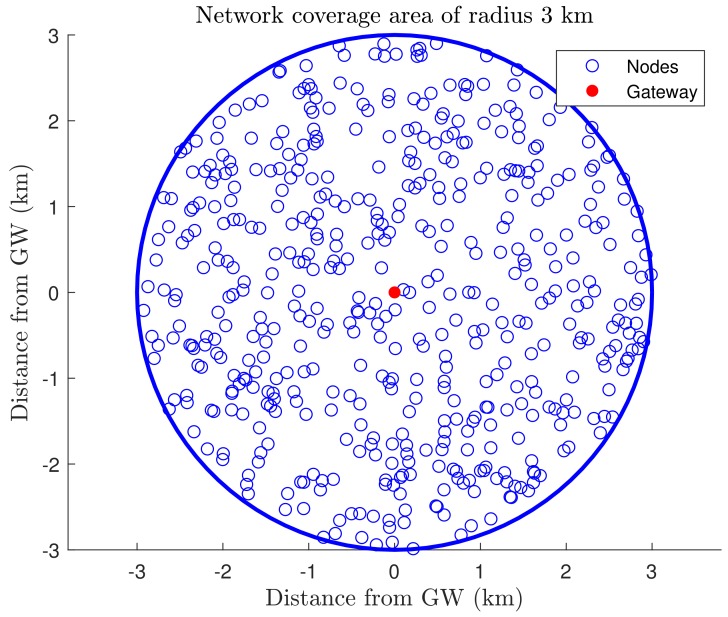
Uniform distribution of $\bar{N}=500$ nodes in a circular network area of radius R=3 km, with the gateway (GW) at the origin.

We consider $\bar{N}$ uniformly distributed smart devices inside an uplink class-A LoRaWAN network without retransmissions, utilizing a single channel within radio range of *R* km and a circular area of $V=\pi R^{2}$ around a single gateway. Figure 1 illustrates a deployment with $\bar{N}=500$ and R=3 km. The gateway is at the origin, and nodes are distributed uniformly in V=28.26 km^2^. Note that such model captures the characteristics of telemetry applications such as those in smart cities and smart buildings. For instance, the University of Oulu Smart Campus has a LoRaWAN network constantly monitoring several sensors such as temperature, luminosity, and $CO_{2}$ [37].

The LoRa modulation bit rate is defined as [14]

(1) $$R_{b}=\frac{4}{4+CR}\frac{BW}{2^{SF}},$$

where $\frac{4}{4+CR}$ is the effective coding rate, ranging from $\frac{4}{5}$ to $\frac{4}{8}$, while CR denotes the LoRa coding rate configuration, varying from 1 to 4. In our work, we assume CR=1, and the LoRa uplink channel aggregated bit rate is expressed as $bitrate_{U}=\sum_{i=7}^{12}Rb_{i}=12.17$ kbps. For instance, Table 1 shows the characteristics of 9 byte LoRa packets with explicit header and CRC modes enabled and BW=125 kHz.

### 3.1. Uplink Outage Probability

The uplink transmission of nodes is based on the ALOHA protocol, and the probability of collision in ALOHA networks is high when many stations are connected [38]. In LoRa, simultaneous signals of different SFs are quasi-orthogonal because the inter-SF rejection gain varies from 16 to 36 dB [39]. Therefore, for the sake of simplicity, our work does not inspect inter-SF interference and focuses on co-SF interference only, which is stronger.

In this paper, the uplink model includes the influence of Rayleigh fading and path loss attenuation as the baseline model [19] for performance analysis, where $g\left(d_{k}\right)=\frac{\lambda }{{\left(4\pi d_{k}\right)}^{\eta }}$ is the path loss attenuation function, η≥2 is the path loss exponent, λ is the wavelength, and $h_{k}$ is the fading in the link between the *k*-th node and the gateway. Let us consider the transmitted signal of a single LoRa node $s_{1}\left(t\right)$ to examine the impact of co-SF interference originated due to simultaneous transmission of nodes with same SF. The mathematical expression of the received signal at the gateway can be expressed as

(2) $$r_{1}\left(t\right)=g\left(d_{1}\right)h_{1}*s_{1}\left(t\right)+\sum_{k=2}^{N}\chi_{k}^{SF}\left(t\right)g\left(d_{k}\right)h_{k}*s_{k}\left(t\right)+n\left(t\right),$$

where $n\left(t\right)$ is additive white Gaussian noise with zero mean and variance $N=-174+NF+10\log_{10}\left(BW\right)$ dBm, NF is the noise figure of the receiver, and −174 dBm/Hz is the thermal noise spectral density constant.

We consider that an outage of the received signal in an uplink channel can take place in the two scenarios [19]. First, if the signal-to-noise ratio (SNR) of the received packet is less than the SF specific threshold $q_{SF}$, then the node is considered disconnected. Second, if the signal-to-interference ratio (SIR) between the target-received packet and any other concurrent signals of the same SF and frequency channel is less than 6 dB, then it is considered as a collision.

#### 3.1.1. Outage Condition I

The distance of the end-device to the gateway in a wireless transmission domain is crucial. The instantaneous SNR can be expressed as $SNR=\frac{P_{1}{\left|h_{1}\right|}^{2}g\left(d_{i}\right)}{N}$, where $P_{1}$ is the transmit power of end-device 1 in mW and $|h_{1}{|}^{2}$ is the squared envelop of the channel coefficient. Communication is only possible when the SNR of the received signal at the gateway is less than the reception threshold $q_{SF}$. Thus, the first outage condition, the connection probability, is defined as [19]

(3) $$H_{1}=exp\left(\frac{Nq_{SF}}{P_{1}g\left(d_{1}\right)}\right),$$

where $d_{1}$ (in meters) is the distance of the desired end-device from the gateway.

#### 3.1.2. Outage Condition II

A collision in LoRa end-device transmission takes place if the SIR of the desired signal with respect to interference from the same SF and frequency channel is less than 6 dB, i.e., if the desired signal is at least four times stronger than the interference. We model this outage condition based on [19], where interference is approached by considering the strongest interfering device. According to [19], the highest interference comes from the end-device $k^{*}$.

The probability that no collision occurs or that the strongest interfering signal is at least 6 dB below the desired one, termed the capture probability, is

(4) $$\begin{array}{cc} Q_{1} & =P\left[\;\frac{|h_{1}{|}^{2}g\left(d_{1}\right)}{|h_{k^{*}}{|}^{2}g\left(d_{k^{*}}\right)}\geq 4\;|\;d_{1}\;\right]=E_{|h_{1}{|}^{2}}\left[\;P\left[\;X_{k^{*}}<\frac{|h_{1}{|}^{2}g\left(d_{1}\right)}{4}\;|\;|h_{1}{|}^{2},d_{1}\;\right]\;\right]. \end{array}$$

The probability above depends on the distribution of $X_{k^{*}}={\left|h_{k^{*}}\right|}^{2}g\left(d_{k^{*}}\right)$. The cumulative distribution function (CDF) of $X_{k^{*}}$ is derived in [19] and is denoted as $F_{X_{k^{*}}}$. Thus,

(5) $$\begin{array}{cc} Q_{1} & =E_{|h_{1}{|}^{2}}\left[F_{X_{k^{*}}}\left(\frac{|h_{1}{|}^{2}g\left(d_{1}\right)}{4}\right)\right]=\int_{0}^{\infty }e^{-z}F_{X_{k^{*}}}\left(\frac{zg(d_{1})}{4}\right)dz. \end{array}$$

Moreover, in [19] the authors present an approximation for (5) that is only accurate at the edges of each annulus. This paper considers only the exact probability in (5).

#### 3.1.3. Coverage Probability

The probability that defines whether a selected end-device is in coverage and can successfully communicate with the gateway is termed the coverage probability. It is the product of $H_{1}$ and $Q_{1}$. The average coverage probability ${℘}_{c}$ can be achieved by deconditioning the location of the individual node by averaging over the network coverage area $V=\pi R^{2}$, i.e., [19]

(6) $${℘}_{c}=\frac{2}{R^{2}}\int_{0}^{R}H_{1}\left(d_{1}\right)Q_{1}\left(d_{1}\right)d_{1}\;dd_{1}.$$

The average coverage probability of a individual SF annulus is also inspected. It indicates the probability of an end-device at distance $d_{1}$ in the annulus *i* by considering the connection and capture probabilities and is defined as [20]

(7) $${℘}_{c,i}=\frac{2}{{\left(l_{i+1}-l_{i}\right)}^{2}}\int_{l_{i}}^{l_{i+1}}H_{1}\left(d_{1}\right)Q_{1}\left(d_{1}\right)\left(d_{1}-l_{i}\right)\;dd_{1},$$

where $l_{i+1}$ is the radius of the outer circle and $l_{i}$ is the radius of the inner circle of the *i*th annulus.

## 4. Proposed SF Allocation Algorithm

In this paper, we propose an SF allocation algorithm, i.e., an algorithm to define the range of each SF annulus. Our solution uses the K-means machine learning algorithm [40], used in the process of vector quantization in data mining by clustering. It is a non-deterministic, numerical, and iterative approach. The main objective of the K-means algorithm is to find the minimum cost function, defined as the distance between each point in the data set and its nearest centroid. The distance between the cluster centers and data elements typically assumes the Euclidean distance. K-means clustering method can efficiently achieve robust clustering results when dealing with large data sets. The K-means algorithm first arbitrarily chooses *K* points from the data set, which indicate the initial centroids. The remaining points are then clustered to the closest centroid, and the coordinates of centroids are recalculated, iteratively, until the cost function converges.

Consequently, it is important to choose the appropriate number of centroids during the initialization procedure because the area of each annulus $\pi (l_{i+1}^{2}-l_{i}^{2})$ increases towards the higher SFs in a strategy based on equal distance steps per SF, which results in the growth of node density due to uniform distribution. That is why it is essential to select the sequences for K-means iterations that can provide larger values of *K* clusters for higher SFs. In order to avoid an extensive number of nodes in an individual SF, there should be a fair difference between the inner ($l_{i})$ and outer ($l_{i+1}$) radii of annulus. In the proposed work, the annulus area is directly dependent on the difference between the *K* clusters for two consecutive iterations.

In our approach, we use five iterations of K-means. We start by computing the boundaries of the outermost SF ring, SF12, and then proceed to define the inner boundaries for lower SFs. For each iteration, *K* clusters are selected to develop the centroids of end-devices in the LoRa network covered by a single gateway. Four different mathematical sequences listed in Table 2—a Fibonacci series, square numbers, Wythoff array, and arithmetic series—are used to assign the values of *K* for each iteration.

In our work, K-means operates iteratively. Each iteration defines the set of nodes at the outer SF ring. In each iteration, the algorithm seeks the set of *K* centroids *C* that minimizes the average of the distances between any node and its closest centroid, i.e.,

(8) $$\begin{array}{c} C=\underset{C_{k}\in C}{\arg\;\min}\;\frac{1}{|ED_{k}|}\sum_{X_{i}\in ED_{k}}dist{\left(C_{k},X_{i}\right)}^{2}, \end{array}$$

where $ED_{k}$ is the set of devices at the *k*-th iteration, $X_{i}$ is a device in $ED_{k}$, and $C_{k}$ is the closest centroid of $X_{i}$. The function $dist(x_{1},x_{2})$ computes the Euclidean distance between $x_{1}$ and $x_{2}$. This procedure returns the collection of *K* centroids of network nodes, whereas $C=\{C_{1},\ldots C_{K}\}$. After computing the centroids, the algorithm determines the boundary of *C*, so that $\left[C_{x},C_{y}\right]=boundary\left(C\right)$, which determines the 2-D vector of border points around the Cartesian coordinates of the centroids. Then, it separates the nodes that are inside of the centroid boundary, forming the set $I=[I_{x},I_{y}]$, where $I_{x}$ and $I_{y}$ are vectors storing the coordinates of the inner nodes in each of the Cartesian dimensions. In the next step, the maximum absolute value of each dimension of *I* is calculated to set the radius as $l_{i}=\frac{max|I_{x}\left|+max\right|I_{y}|}{2}$, which defines the limit of the SF ring *i*. The procedure repeats to determine the boundaries of the remaining SF rings ($l_{5},\ldots l_{1}$).

The steps involved in the proposed SF allocation technique are described in Algorithm 1. It repeats the process five times to allocate nodes for SF12–SF8.At the end, the remaining nodes use SF7. Initially, the SF12 outer limit is set to the network radius. In each iteration, the number of clusters is assigned to *K* depending on the chosen mathematical series (as mentioned in Table 2). For the first iteration, the algorithm considers all of the $\bar{N}$ nodes inside the set ED. In line 4, it computes the K-means of ED, which returns the centroids $C=\{C_{1},\ldots C_{K}\}$ by (8). Since nodes in the set *I* are inside the boundary of centroids, line 7 computes the inner limit of SF ring $l_{i}$. This process is repeated iteratively until $l_{1}$ is calculated for the allocation of SF8 and the remaining nodes are assigned to SF7 (line 11). Note that the set of nodes ED is updated at the end of each iteration by removing the nodes that were already allocated to an SF (line 9).

**Algorithm 1** K-Means-based SF Allocation
**Input:** ED: = $\bar{N}$ uniformly deployed nodes
**Output:***L*: = $\left\{l_{0},l_{1},\ldots ,l_{6}\right\}$
1: $l_{6}$: = *R*2: **for** i **in**
{5,…,1}**do**▹ For each SF ring, starting from the outermost ring3:     *K*: = GetKfromSeries(i)▹ Set number of centroids for this iteration4:     *C*: = Kmeans(ED, *K*)▹ Compute the centroids5:     *B*: = boundary(C)▹ Compute the boundary of *C*6:     *I*: = {x∈ED|x∈convB}▹ Select nodes that are inside the boundary *B*7:     $l_{i}:=\frac{max(|I_{x}\left|\right)+max(|I_{y}\left|\right)}{2}$▹ Compute the new SF ring limit8:     $SF_{i+7}$: = $\left\{x\in ED\;|\;x\notin Ball\left[\left(0,0\right),l_{i}\right]\right\}$▹ Allocate $SF_{i+7}$ to nodes outside the circle of radius $l_{i}$9:     ED: = $\left\{x\in ED\;|\;x\in Ball\left[\left(0,0\right),l_{i}\right]\right\}$▹ Remove nodes outside the circle of radius $l_{i}$10: $l_{0}$: = 011: $SF_{7}$: = ED▹ Allocate $SF_{7}$ to remaining nodes12: **return** L

**Figure 2:**
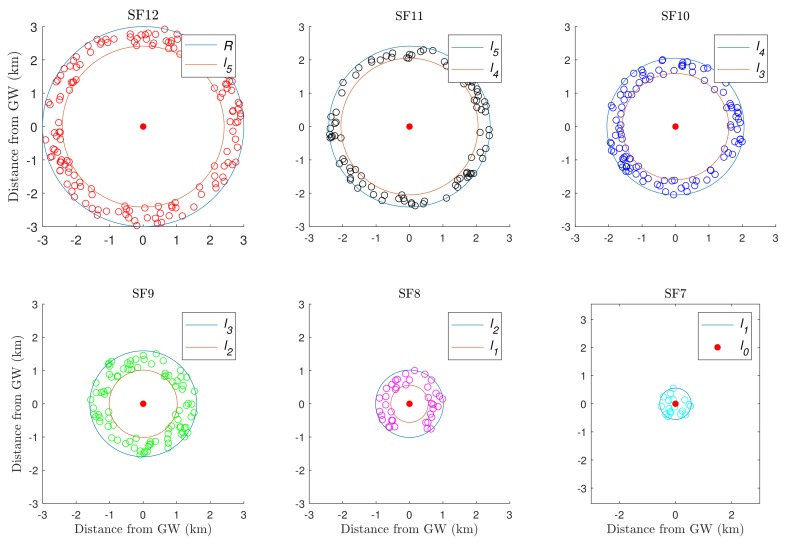
K-means iterations for SF allocation based on Fibonacci series and 500 nodes, where $l_{i}$ and $l_{i+1}$ are the inner and outer radii of *i*^th^ annulus, respectively.

All of the iterations of the proposed algorithm for an example network are demonstrated in Figure 2. The radius of the network circular area is R=3 km, and therefore, the outer limit of SF12 is $l_{6}=R=3$ km. The first iteration of the algorithm defines the inner boundary of SF12, $l_{5}$, as shown in Figure 2. After excluding the devices inside the SF12 ring from $ED_{k}$, the algorithm runs a new iteration and defines $l_{4}$, i.e., the inner boundary of the SF11 ring. The iterations continue until $l_{1}$ is defined and the complete network geometry is obtained.

**Figure 3:**
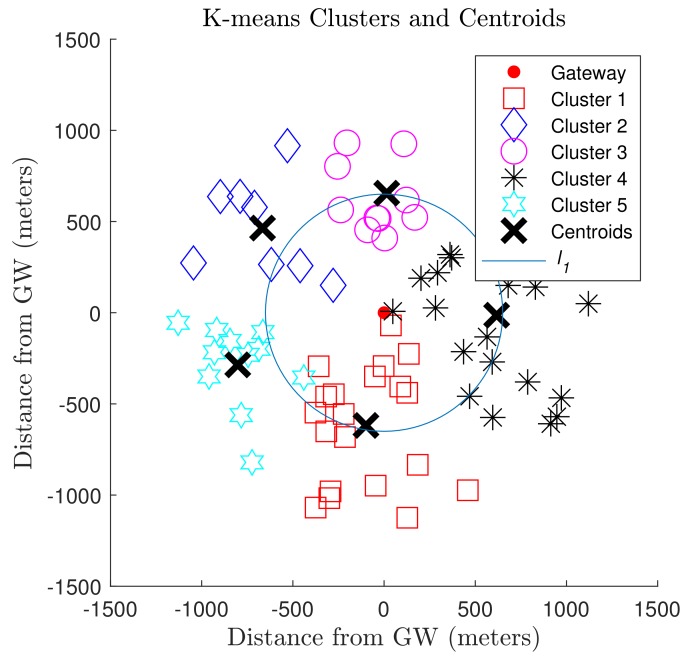
5th iteration with Fibonacci series. Nodes outside $l_{1}$ use SF8, those inside use SF7.

**Figure 4:**
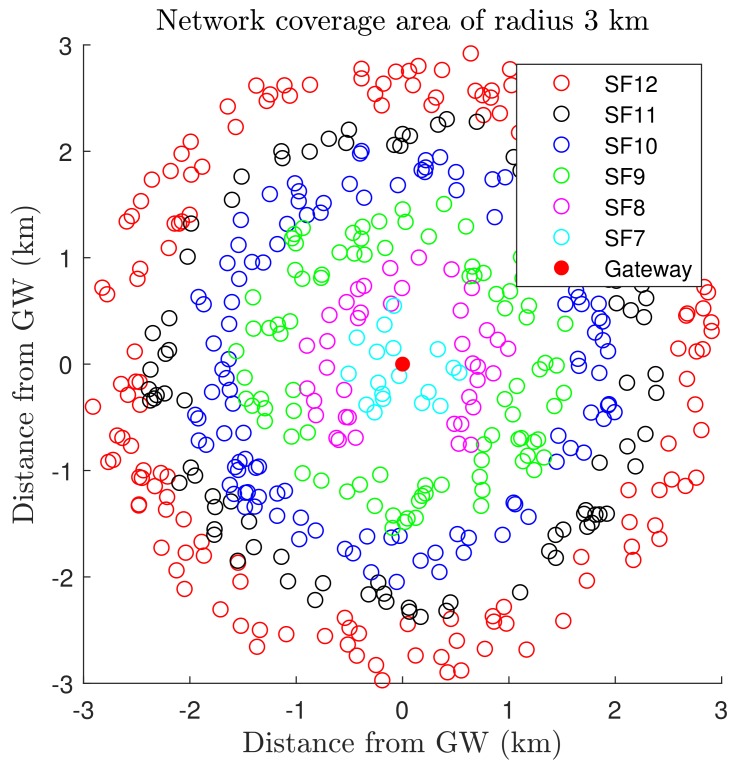
SF allocation of 500 nodes with Fibonacci series.

Figure 3 depicts the clusters of nodes, centroids, and gateway at the origin for the last K-means iteration. The nodes outside $l_{1}$ are allocated to SF8, and the remaining nodes are assigned to SF7. The SF distribution of $\bar{N}$ = 500 nodes based on the proposed approach considering the Fibonacci series for clustering is shown in Figure 4. The number of nodes in SF rings depends on the chosen series because of the distinct number of clusters for each sequence.

The area of each annulus also varies according to the mathematical series, which also affects the network performance. An important aspect to take into consideration is the selection of the number of clusters (*K*); if the difference between the clusters of two consecutive iterations is too big, that will result in a large number of end-devices in that specific region and, as a consequence, the probability of collisions and of co-SF interference will be high. In the same way, SF7 will have a larger coverage area and more nodes if *K* is high for the last iteration.

## 5. Numerical Results and Discussion

In this section, we evaluate the scalability and performance of the proposed methodology by means of computer simulations. The results are based on a $p_{0}$ = 1% duty cycle, BW=125 kHz, η = 2.75 path loss exponent, 868 MHz European frequency band, and network radius of R=3 km. Table 3 summarizes the parameters considered for the results.

### 5.1. SF Allocation and Scalability Analysis

As discussed in Section 4, we used mathematical series to assign the numbers of clusters for K-means iterations. The Fibonacci series has the shortest ranges for SF7, 715 m for $\bar{N}$ = 500. The distance between the SFs and the distribution of nodes can be changed by modifying the number of clusters (*K*). On the other hand, for the same number of nodes, the Square series has a longer range for SF7, which is 1201 m, and it contains more end-devices. This type of configuration is due to the higher value of *K* clusters for iterations (see Table 2). While in the case of Fibonacci and equal-distance-based SF allocation in [19,20], the network has fewer nodes in SF7, and the number of nodes in each SF region increases considerably towards the higher SFs, as shown in Table 4. The average SF ranges for the Fibonacci series, square series, Wythoff array and arithmetic series are shown in Table 5.

**Figure 5:**
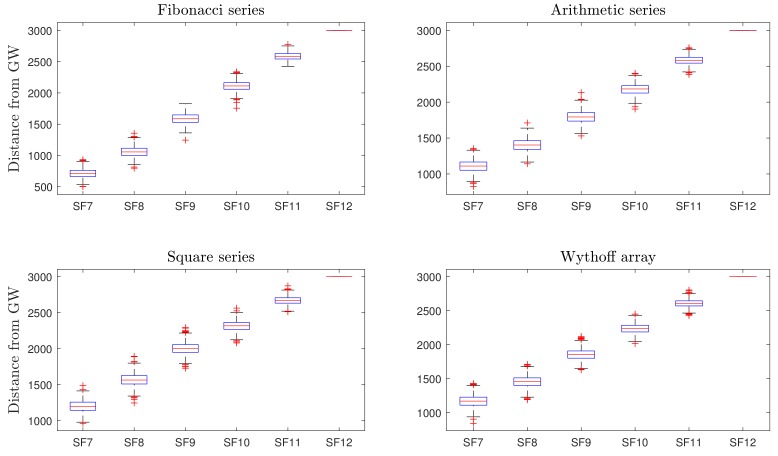
Boxplots representing the distance of SFs from the gateway. The red “+” signs indicate outliers. SF12 has a constant distance of 3 km for all of the series.

The boxplot is a standard process to quantify the variability of data on the basis of five parameters, i.e., the minimum, first quartile (25%), median, third quartile (75%), and maximum. Th distance of the SFs boundary from the gateway for $\bar{N}$ = 500 is demonstrated in Figure 5 for each of the considered series.

The median of every SF is identical to distances provided in Table 5. As depicted in the graphical results, SF7 and SF8 have a large disparity in range and number of nodes for different scenarios, while SF11 and SF12 have nearly close coverage areas for all scenarios. Furthermore, we can also clearly observe that variation in *K* clusters selection has a direct effect on SF allocation based on the proposed methodology. The number of nodes in each SF are illustrated in Table 4. Square-series-based networks demonstrate five times more nodes in SF7 as compared to the reference model.

The large values of *K* clusters for the last iterations result in longer radii that directly increase the region of SF7 and keep SF8 further away from the gateway. These different scenarios can be used according to different situations and requirements of LoRa applications. An approach based on the Fibonacci series is beneficial for applications where fewer nodes in lower SFs are required, while the Wythoff array, square series, and arithmetic series have wider regions for SF7, and thus can be used in setups where more nodes are required in SF7 inside the radio range of nearly 1200 m to provide highest data rate ($R_{b}$ = 5.47 kbps, see Table 1). Several studies examined the performance of LoRa networks and show that the success probability of data packets decreases for higher SFs. In our work, we consider the effect of changing the coverage range and varying the number of devices for individual SFs.

### 5.2. Performance Analysis

**Figure 6:**
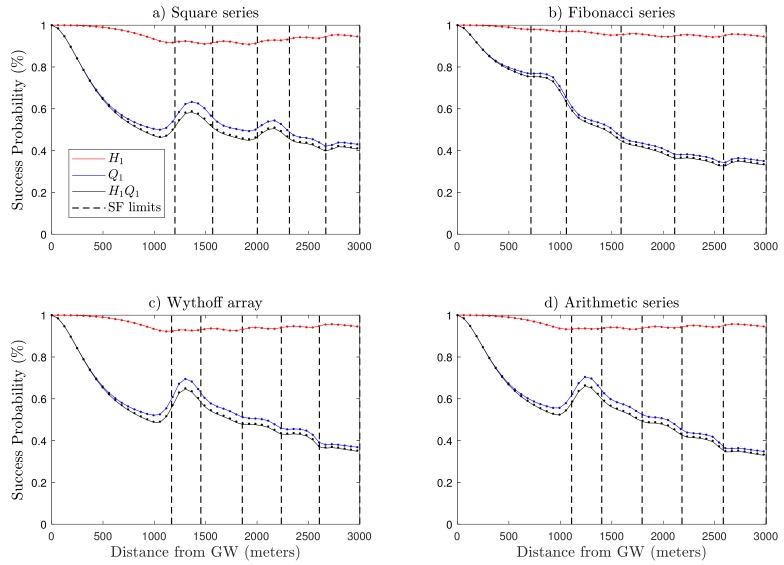
LoRa uplink performance with the proposed SF allocation algorithm for different series and $\bar{N}$ = 500 nodes. (**a**) Square series with K={49,36,25,16,9}. (**b**) Fibonacci series with K={34,21,13,8,5}. (**c**) Wythoff with K={37,32,24,16,11}. (**d**) Arithmetic series with K={34,28,22,16,10}.

After the application of the proposed SF allocation algorithm, we investigated the performance of the resulting LoRa network. The theoretical results were verified by Monte Carlo simulations. In the figures, each marker represents the average over $10^{5}$ random deployments of the Poisson point process (PPP) for a single gateway LoRa uplink model, considering an end-device at $d_{1}$ meters from the gateway. In Figure 6, the solid lines demonstrate the theoretical results, while marker points of the same color illustrate the simulation outputs. The simulated results align with the theoretical ones. Within the context of the previously discussed mathematical sequences, we considered and examine the impact of different SF allocation scenarios on connection probability $H_{1}$, capture probability $Q_{1}$, and coverage probability $H_{1}$$Q_{1}$ against the distance from the gateway.

As expected, the distance of the end-device from the gateway has considerable influence on connection probability $H_{1}$. In the case of the Fibonacci series, the model has a better success probability for lower SFs as compared to the square series, arithmetic series, and Wythoff array. This fact is due to the difference in a range of individual SF boundaries for said SF allocation schemes and the distance-dependent SNR threshold $q_{SF}$. Furthermore, in the scenario of the square series, SF7 has large coverage areas of 1201 m (see Table 5), which affects path loss attenuation and the instantaneous SNR. The SNR threshold $q_{SF}$, however, remains the same at −6 dB (see Table 1). As a consequence, the outage condition in (3) slightly degrades the network connection probability. Although a performance boost is illustrated during transitions of end-devices into the next SF because of the lower value of $q_{SF}$, the performance of the previous SF has a direct consequence on the next SF.

**Figure 7:**
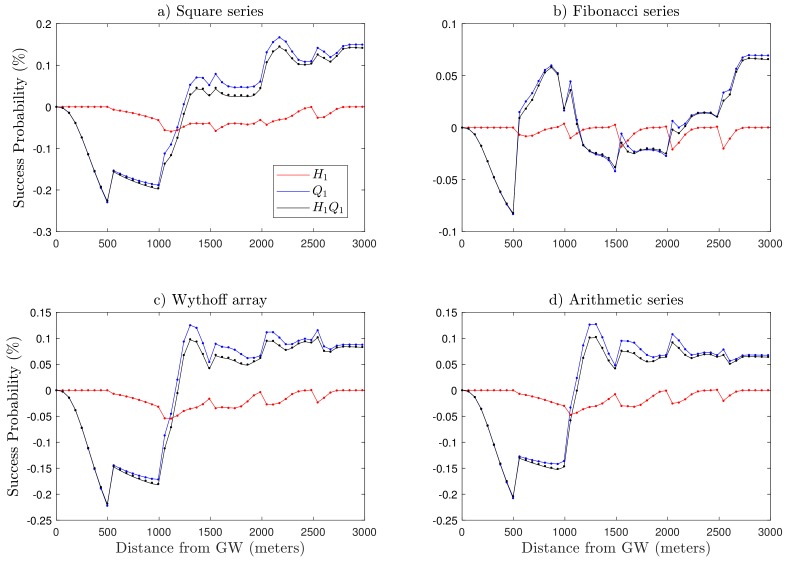
Comparison of performance gain of different series with respect to the baseline model for $\bar{N}$ = 500, network size of *R* = 3 km with $p_{0}$ = 1%, and path loss exponent η = 2.75.

Moving towards the capture probability $Q_{1}$, unlike $H_{1}$, it considers co-SF interference. $Q_{1}$ declines gradually with increasing SF, as illustrated in Figure 6. This trend is because of two major factors including ToA and the number of nodes in each annulus. ToA grows exponentially with SF, thus for the higher SFs, the wireless channel remains occupied for a long time slot, which increases the risk of collisions between simultaneously transmitted LoRa packets. In the same way, the number of end-devices in an individual annulus increases for higher SFs due to the uniform distribution of nodes in the circular coverage area, as demonstrated in Table 4. As a result, the network experiences co-SF interference that degrades the quality of transmission. For the cases with the square series, arithmetic series, and Wythoff array (Figure 6a,c,d), the model has a larger coverage area and more nodes in SF7, resulting in higher co-spreading factor interference, which is the major reason behind the lower performance of the network for these specific scenarios. Although we are sacrificing network quality for lower SFs with fewer nodes and high success probabilities, as presented in Figure 7, we improved the performance of the network for the higher SFs and regions with more nodes, where the network performance was weak in the baseline model from [19], which considers fixed distance steps from the gateway to define the SF allocation. Moreover, SF allocation based on the square series (Figure 6a) has better network performance, which happens because of improved gain in $Q_{1}$ for higher SFs as compared to the Fibonacci series, arithmetic series, and Wythoff array.

**Figure 8:**
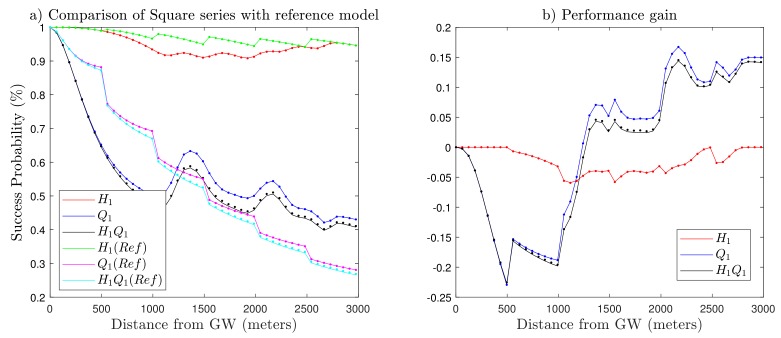
(**a**) Performance comparison of square series with the reference model for $\bar{N}$ = 500. (**b**) Performance gain (success probability of proposed model − success probability of reference model). The proposed SF allocation approach sacrifices $Q_{1}$, $H_{1}Q_{1}$ for the lower SFs but achieves greater performance for the higher SFs.

In Figure 8, we present the performance of the square series based on the proposed SF allocation algorithm in comparison with the baseline model. First, we observe that the capture probability $Q_{1}$ and coverage probability $H_{1}$$Q_{1}$ of the baseline model outperform the proposed algorithm in the region of radius R>1000 m from the gateway. Here, it is worth mentioning that there are fewer nodes in this region as compared to the remaining area of the network. The proposed algorithm surpasses the baseline model with a gain in capture probability $Q_{1}$ (up to 53% for SF12 in Figure 8b). In addition, the nodes closer to the gateway always have better behavior in contrast with nodes far away, so that the network can tolerate a lower success probability with a boost in performance of farther end-devices. As expected, there was more change in success probability in the higher SFs region as compared to baseline model because of the fair distribution of SF by the proposed algorithm. Figure 8b shows the difference between the baseline model and the square series in terms of outage probabilities through the course of distance. The zero level on the y-axis (success probability) shows no difference, while there is a positive/negative gain either on the upper or lower side of that level. There is up to 16.73% growth in $Q_{1}$ demonstrated by the end-devices present in higher SFs.

**Figure 9:**
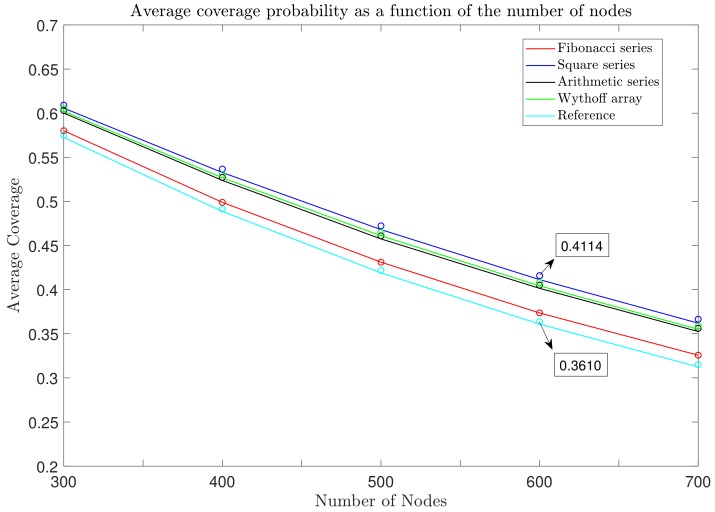
Comparison of the coverage probabilities of the LoRa uplink as a function of the number of nodes ranging from $\bar{N}$ = 300 to 700 nodes for the network size of *R* = 3 km with $p_{0}$ = 1% and path loss exponent η = 2.75.

Moreover, we also consider the evaluation of the average coverage probability of the networks for different numbers of nodes ($\bar{N}$) ranging from 300 to 700, and results demonstrate that ${℘}_{c}$ drops exponentially towards higher $\bar{N}$. Figure 9 depicts the average coverage probability of different numbers of end-devices. The SF allocation schemes deployed using the square series demonstrated a better performance gain than all other scenarios, including the reference model. It was followed by the Wythoff array, arithmetic series, and Fibonacci series, in that order. The proposed SF allocation scheme overcomes the performance of the baseline model by an overall growth of around 5% in its ${℘}_{c}$. For instance, taking the square series into account, at $\bar{N}$ = 500 there is a boost in the average coverage probability (6) from ${℘}_{c}$ = 41.9% to 46.81% compared with the reference model. On the other hand, SF allocation schemes deployed using the Fibonacci series showed the least-improved network coverage probability compared to all other user distribution series.

**Figure 10:**
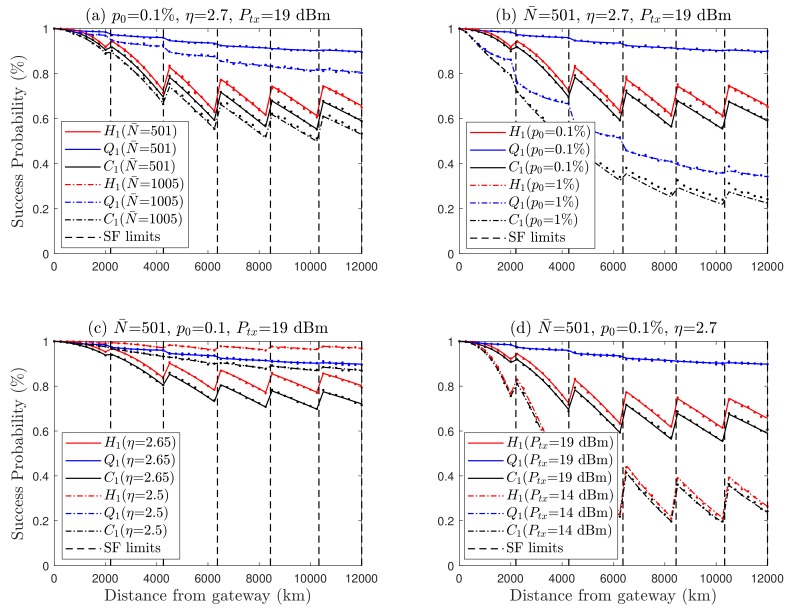
Performance LoRa uplink considering Fibonacci series K-means clustering, and the impact of different parameters on success probabilities. (**a**) Density of users increased from $\bar{N}$ = 501 to $\bar{N}$ = 1005. (**b**) Duty cycle increased from $p_{0}=0.1\%$ to $p_{0}=1\%$. (**c**) Path loss exponent from η=2.5 to η=2.65. (**d**) Transmit power from ($P_{tx}=14$) dBm to $P_{tx}=19$ dBm.

We also investigated the performance of the proposed model taking into account the variation of different parameters on Fibonacci series. As seen in Figure 10a,b, $H_{1}$ is agnostic to the number of nodes and duty cycle. It is assessed at 0.1% and 1%, which is within the duty cycle range specified by ETSI for LoRa applications [34,35]. Furthermore, node density and the duty cycle demonstrate a negative impact on $Q_{1}$ because of co-SF interference caused by increasing medium usage from $\bar{N}=501$ nodes to $\bar{N}=1005$ nodes. Likewise, in Figure 10c, the path loss exponent η illustrates network connection degradation at 2.65 from 2.5 because $H_{1}$ depends on the distance, while the the capture probability ($Q_{1}$) is not dependent on the path loss exponent. In the case of one frequency channel, the transmit power $P_{k}$ of nodes can be up to 20 dBm (100 mW) [11,41]. In order to evaluate the effect of different transmit powers, in Figure 10d we raised the transmit power from 14 dBm to 19 dBm. The results demonstrate that the transmit power of 19 dBm causes a better connection probability as compared to 14 dBm. Nevertheless, variations of the path loss exponent and transmit power do not affect $Q_{1}$ considerably because it is much more dependent on the number of nodes.

### 5.3. Discussion

The architecture of LoRaWAN consists of end-devices, a gateway, network server (NetServer), and application server [15]. The NetServer is mainly responsible for the overall management of the network. The dynamic configuration of the SF by the NetServer is already possible in LoRaWAN during the network join procedure or through specific MAC commands. In fact, these features are used in LoRaWAN when the adaptive data rate (ADR) mechanism is active. For our approach to be implemented in practice, the NetServer could run our algorithm periodically or when the number of connected devices changes significantly, and then issue the required MAC commands to reconfigure the devices that need to change their SF. Therefore, as the current LoRaWAN specification is already able to dynamically allocate SFs, our proposal only changes the way the proper allocation is calculated at the NetServer, and is therefore feasible in practice.

## 6. Conclusions

This paper has presented a novel SF allocation technique for a large-scale LoRa network using the K-means clustering machine learning algorithm. The authors also analyzed the impact of the distance of end-devices from the gateway and the number of nodes in each SF on network performance. In this work, four different scenarios are considered, which have different distances for the SF boundaries and variations in the number of nodes in an individual SF. Such fair distribution results in a better average coverage probability in the higher SFs, while dealing with the maximum number of nodes. Numerical findings show that our SF allocation algorithm outperforms the reference model not only in terms of success probability but also in regards to fair resource distribution. The evaluated theoretical and simulation results are useful for an in-depth understanding of large and dense LoRa networks. Our resource allocation method can handle dense and large circular coverage areas for LoRa sensors using distinct numbers of clusters instead of equal-radius-based SF allocation [19,20], while the techniques in [28,29,33] are designed for short-range networks. The studies [19,20,28,29,33] only highlighted the performance of networks for fixed parameters. In contrast to them, our work inspects different scenarios by obeying the restrictions of ETSI standards.

## Figures and Tables

**Table 1:** Characteristics of the LoRa uplink model containing packets of 9 bytes at BW = 125 kHz.

SF (i)	Bit Rate kbps (${\mathrm{Rb}}_{i}$)	Receiver Sensitivity dBm	SNR dB ($q_{\mathrm{SF}}$)	Range km
7	5.47	−123	−6	$l_{0}-l_{1}$
8	3.13	−126	−9	$l_{1}-l_{2}$
9	1.76	−129	−12	$l_{2}-l_{3}$
10	0.98	−132	−15	$l_{3}-l_{4}$
11	0.54	−134.5	−17.5	$l_{4}-l_{5}$
12	0.29	−137	−20	>$l_{5}$

**Table 2:** *K* Cluster Values for K-Means Iterations.

Iteration Series	1st SF12	2nd SF11	3rd SF10	4th SF9	5th SF8
**Fibonacci series**	34	21	13	8	5
**Square number**	49	36	25	16	9
**Arithmetic series**	34	28	22	16	10
**Wythoff array**	37	32	24	16	11

**Table 3:** System Parameters.

Parameter	Symbol	Value
Nodes	$\bar{N}$	300–700
Spreading Factor	SF	7–12
Bandwidth	BW	125 kHz
Carrier frequency	*f*	868 MHz
Noise figure	NF	6 dBm
Transmit power	$P_{1},$	14 dBm
Duty cycle	$p_{0}$	1%
Path loss exponent	η	2.75

**Table 4:** Comparison of the proposed approach with the reference method, based on the number of nodes.

Series	$\bar{N}$	SF7	SF8	SF9	SF10	SF11	SF12
	300	18	19	48	66	75	77
Fibonacci series	500	29	35	79	107	124	129
	700	40	51	110	148	173	180
	300	44	24	43	52	63	77
Arithmetic series	500	69	42	70	87	107	129
	700	94	61	98	121	149	180
	300	53	33	55	44	58	61
Square numbers	500	81	57	87	75	99	105
	700	111	81	121	105	137	148
	300	49	24	45	52	60	73
Wythoff array	500	77	42	75	87	100	123
	700	103	62	103	121	140	173
	300	9	25	42	59	76	92
Reference model	500	14	42	70	98	126	152
	700	20	59	97	137	176	214

**Table 5:** Comparison of proposed approach with the reference method based on the distance of individual SF outer boundaries from the gateway (values in meters).

Series	$\bar{N}$	SF7	SF8	SF9	SF10	SF11	SF12
	300	735	1049	1586	2112	2588	3000
Fibonacci series	500	715	1060	1591	2112	2586	3000
	700	707	1071	1601	2112	2587	3000
	300	1144	1412	1806	2194	2590	3000
Arithmetic series	500	1110	1403	1795	2183	2584	3000
	700	1099	1409	1801	2188	2588	3000
	300	1248	1591	2037	2336	2680	3000
Square numbers	500	1201	1568	2004	2316	2670	3000
	700	1190	1568	2002	2313	2667	3000
	300	1209	1469	1872	2246	2613	3000
Wythoff array	500	1168	1453	1857	2237	2607	3000
	700	1150	1450	1851	2231	2604	3000
	300	500	1000	1500	2000	2500	3000
Reference model	500	500	1000	1500	2000	2500	3000
	700	500	1000	1500	2000	2500	3000
